# Early Performance of a Multifilament Resorbable Synthetic Mesh in Aesthetic Breast Surgery: A 206 Patient Experience

**DOI:** 10.1093/asjof/ojag109

**Published:** 2026-06-08

**Authors:** Renee M Burke, Charalambos K Rammos

## Abstract

**Background:**

Increasing evidence supports the role of soft-tissue reinforcement in enhancing outcomes and longevity in breast surgery. Maintenance of lower pole shape, soft-tissue support and implant position often requires use of mesh in aesthetic procedures. The authors describe their experience utilizing a fully resorbable multifilament scaffold (TIGR® Matrix, Novus Scientific, Uppsala, Sweden) as an alternative to biologic and synthetic meshes.

**Objectives:**

To assess early safety and performance outcomes when TIGR Matrix is utilized in primary and revision cosmetic breast surgery.

**Methods:**

We began routinely utilizing TIGR matrix in aesthetic breast surgeries in 2023 and retrospectively reviewed all patients who underwent primary or revision elective cases with the mesh. Indication for use and examination of product performance was performed via a retrospective review.

**Results:**

TIGR matrix was used in 206 patients (407 breasts), mean age 42.6 years, with mean follow-up of 9.2 months. Mean BMI was 24.2 with 30 patients (14.6%) having a history of massive or major weight loss. One hundred fourteen cases (55.3%) were primary procedures, 92 (44.7%) were revisions. Mesh was placed in the subfascial position in 97 (47.1%) cases and submuscular in 109 (52.9%). Ten patients (4.9%) required postoperative intervention; 5 seromas in 4 patients (1.9%) required mesh removal and implant exchange and 6 patients required revision surgeries due to animation deformity (5) and implant displacement (1).

**Conclusions:**

Use of TIGR matrix demonstrated early safety and effectiveness, achieving consistent clinical outcomes in both primary and revision aesthetic breast surgeries.

**Level of Evidence: 3 (Therapeutic):**

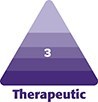

Achieving long-lasting, breast shape and contour is the primary goal of aesthetic breast surgery. However, the quality and strength of native soft tissue are often insufficient to provide long-term support. This challenge is especially evident in revision procedures, patients with thin tissue or those desiring large implants, predisposing to complications such as ptosis, implant malposition, stretch deformity, and overall aesthetic dissatisfaction.^1,2^

Acellular dermal matrices (ADMs) have historically provided additional pocket support and improved implant control but are associated with high cost and variable biologic properties.^3-5^ Synthetic meshes offer an alternative that is more cost-effective, without the inherent variability of biologic materials.

Permanent synthetic meshes have been used to provide durable soft-tissue reinforcement in breast surgery, particularly in reconstructive settings; however, their use in aesthetic procedures has been limited. Concerns inherent to their nonresorbable design include chronic foreign-body response, palpability, and long-term material deformity.^6^ To address these shortcomings, synthetic absorbable meshes have been developed to provide temporary mechanical support during healing while allowing gradual load transfer to native tissue as the scaffold resorbs.

Resorbable mesh profiles vary with scaffolds constructed of poly-4-hydroxybutyrate (P4HB) designed for resorption to occur over approximately 18 to 24 months. In vivo reports describe the material as supporting tissue remodeling with minimal chronic inflammation on histologic evaluation and clinical experience in aesthetic breast surgery, including revision augmentation and mastopexy, has shown maintenance of lower-pole support and acceptable complication profiles in short- and mid-term follow-up.^7,8^ Resorbable mesh constructed from polydioxanone (PDO) is designed for more transient reinforcement, with complete resorption reported to occur within 6 to 12 months. Early clinical series using this material have reported low rates of seroma and infection with preservation of soft-tissue support during healing.^9^

TIGR® Matrix is a dual-fiber resorbable synthetic mesh composed of fast- and slow-absorbing polymers designed for staged support over 3 years. This design provides initial reinforcement of the implant pocket and lower pole during healing, followed by progressive transfer of support to the soft tissues as the mesh is resorbed and replaced with tissue remodeling. Clinical studies evaluating TIGR Matrix in implant-based breast reconstruction have demonstrated short-term complication rates comparable to those reported for other absorbable mesh options.^10,11^

The objective of this study was to assess early safety and performance outcomes when TIGR Matrix is utilized in primary and revision cosmetic breast surgery.

## METHODS

This study retrospectively reviewed all adult patients who underwent primary or revision elective aesthetic breast surgery with TIGR® matrix (Novus Scientific, Uppsala, Sweden) performed by 2 solo, board-certified, private practice plastic surgeons using TIGR Matrix between August 2023 and September 2025. Patients were excluded for nicotine use within 2 weeks of surgery, as confirmed by urine testing, or if they were unable or unwilling to return for follow-up. While there was no strict upper cutoff for BMI that excluded patients from this series, <5% (10 women) had a BMI >30. Demographic, procedural, and follow-up outcomes data were obtained from electronic health record reviews.

Preoperative indications for the use of soft tissue support in these cases were reviewed, and clinical scenarios were categorized as prophylactic or therapeutic, based on whether reinforcement was used to reduce the risk of soft-tissue failure in primary surgery or to correct established deformities in revision cases.

Prophylactic indications within primary surgery with augmentation included use of the subfascial plane for augmentation, with the scaffold used to enhance lower-pole support and pocket stability, breast or chest wall asymmetries or deformities, such as footprint and inframammary fold asymmetry, lower pole constriction or congenital abnormalities. Patients presenting with poor soft-tissue quality, for example after massive weight loss, with thin tissue or striae and patients desiring large volume implants (>400 cc) were also indicated for prophylactic soft tissue support with mesh placement.

Therapeutic indications require using soft tissue support during revision surgery to address complications arising from prior procedures, address structural problems, and redefine and reinforce the new pocket. Common therapeutic indications included implant malposition, including symmastia, stretch deformity of the inferior pole soft tissue, inframammary fold disruption, and capsular contracture.

Aesthetic breast procedures with supportive matrices are standard of care for appropriate patients in both practices; thus, all patients in the series provided standard surgical consent and this review was conducted in accordance with the Declaration of Helsinki. This study was reviewed by an independent IRB (Tempus IRB, IRB00014644) and determined to be exempt. Patient data were deidentified, and patients were not contacted or reidentified as part of this review.

### Material

TIGR Matrix is a knitted synthetic mesh made of 2 types of untwisted multifilament fibers, a fast-absorbing fiber and a slow-absorbing fiber, affording support during different phases of wound healing and soft tissue remodeling. The faster absorbing fiber makes up 40% of the scaffold and is a copolymer of glycolide, lactide, and trimethylene carbonate. This fiber degrades between 2 weeks and 4 months following implantation and is designed to provide strength during the granulation phase of wound healing. The slow-absorbing fiber makes up the remaining 60% of the scaffold and consists of lactide combined with trimethylene carbonate. This slower-degrading component, which resorbs over 6 to 36 months, maintains long-term integrity and contributes elasticity to the mesh as the tissues transition from the granulation phase to the remodeling phase. The macroporous scaffold facilitates rapid vascularization and supports a transient inflammatory response that is subsequently replaced by organized collagen deposition. Histologic analysis demonstrates that the collagen deposition resembles native connective tissue, with an increased type I to type III collagen ratio reflecting a mature and structurally stable extracellular matrix.^12^

### Surgical Technique

**Figure 1:**
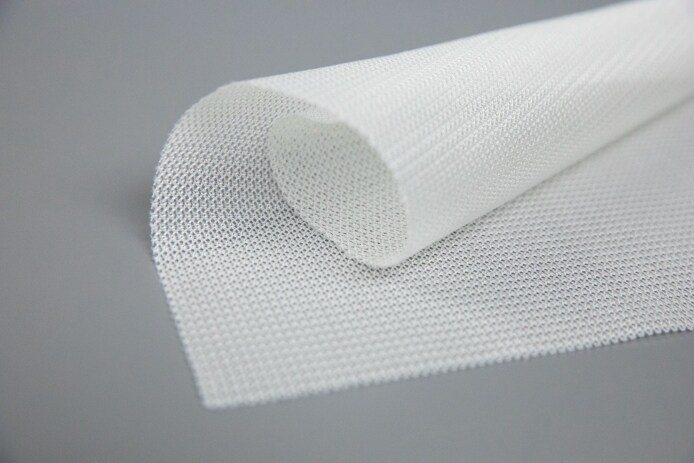
TIGR® Matrix (Novus Scientific, Uppsala, Sweden).

After induction of general anesthesia and standard sterile preparation and draping of the chest, the procedure is performed through standard mastopexy and/or augmentation incisions as indicated. Following development of the breast pocket and intraoperative irrigation with hypochlorous acid for 1 minute, a 15 × 20 cm or 20 × 30 cm TIGR Matrix (Figure 1) is selected and trimmed to the desired size to conform to the inferior pole of the breast, extending from the medial to the lateral inframammary fold and superiorly to the level of the lower pole parenchyma. Care is taken to fashion the mesh to provide broad, tension distributing support without excess bulk or folding.

The mesh is positioned superficial to the pectoralis muscle, covered by the undersurface of the breast and pectoralis fascia for the subfascial approach and secured with 2-4 interrupted 2-0 PDS sutures into the pectoralis muscle and chest wall. For submuscular implants, the mesh rests on the chest wall, partially covered by the pectoralis muscle, and partially covered by the undersurface of the breast, also secured with 2-4 interrupted 2-0 PDS sutures into the perichondrium or periosteum of the rib.

**Figure 2:**
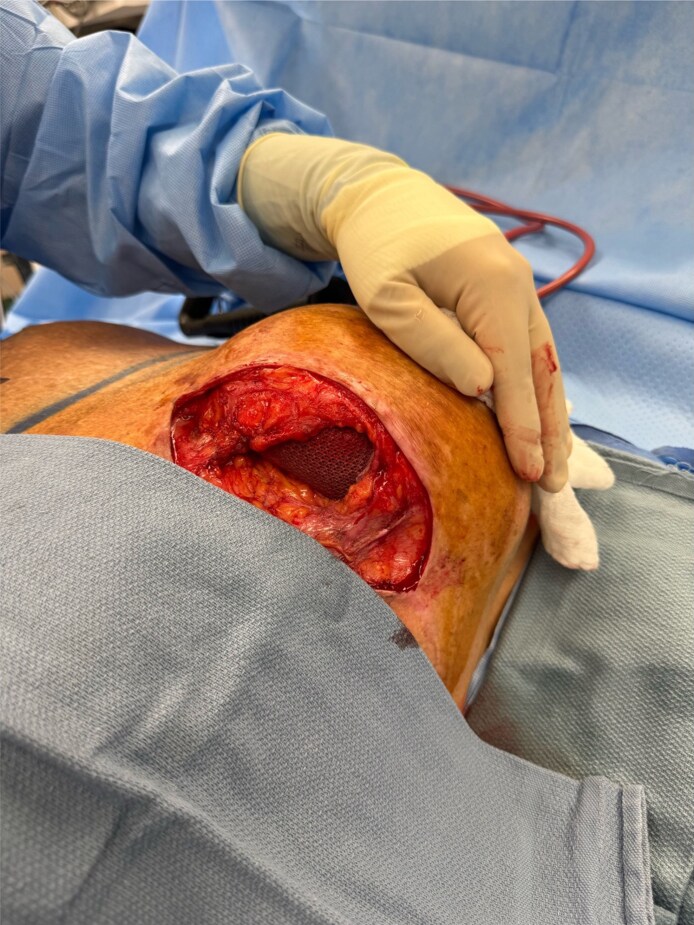
Mesh positioning: intraoperative view of implant with mesh in place, demonstrating lower pole support and pocket stability.

Implants used were either first generation smooth surface silicone gel or sixth generation smooth silk surface silicone gel. In all cases, the mesh provided stable lower pole support, maintained pocket shape, and distributed implant or tissue load **(**Figure 2). Layered closure of breast tissue and skin was performed, and a supportive postoperative bra was used. It is critical to suture the posterior lamella of the breast to avoid any mesh exposure in the case of skin dehiscence.

Our routine postoperative protocol for all patients includes a 1 week course of prophylactic antibiotics, typically cephalexin, doxycline, or bactrim. Patients may also be prescribed 1000 IU Vitamin E for 3 months. For patients presenting with capsular contracture, 10 mg Singulair for 6 weeks is added to their postoperative regimen. Our standard practice is to ask patients to return for follow-up evaluation at 1 week, 2 weeks, 6 weeks, 3 months, 6 months, and annually thereafter. Any follow-up captured in retrospective medical record review was collected.

## RESULTS

A total of 206 women (407 breasts) are included in this review. Patient and operative characteristics are summarized in Tables 1 and 2. Patients’ mean age was 42.6 ± 9.8 years, and mean BMI was 24.2 ± 3.6 kg/m^2^. Thirty patients (14.6%) had a history of significant weight loss. One hundred 14 cases (55.3%) were primary procedures, and 92 patients had previous aesthetic breast surgery. Of the 92 revision cases, 11 (12%) presented with a preoperative diagnosis of Baker grade IV capsular contracture. Average implant volume was 380.6 ± 144.6 cc with large volume implants (>400 cc) used in 100 cases. The mesh was placed in the subfascial position in 97 (47.1%) cases and submuscular in 109 (52.9%). Follow-up and postoperative outcomes are summarized in Tables 3 and 4. Mean follow-up was 9.2 months (range 1-24 months), with 97 patients followed at 1 year or greater timepoints. One patient did not respond to any communications after 6 weeks postsurgery and is considered lost to follow-up. Ten patients (4.9%) required postoperative intervention. These included 5 cases of seroma in 4 patients, which required mesh removal and implant exchange. Additionally, 6 patients required revision surgeries due to animation deformity in 5 and implant displacement in one. There were no instances of postoperative infection, other wound events, or capsular contracture observed.

**Table 1:** Patient Characteristics

	Total (*N* = 206 patients)	
Variables	*n*	%
Age (SD)	42.6 (9.8)	
BMI (SD)	24.2 (3.6)	
Diabetes	1	0.5
Hypertension	16	7.8
History of massive/major weight loss	30	14.6
Depression	34	16.5
Anxiety	29	14.1
Ethnicity		
Caucasian	187	90.8
Non-Caucasian	19	9.2

**Table 2:** Operative Characteristics

Variables	*n*	%
Type of surgery		
Primary	114	55.3
Augmentation	30	26.3
Mastopexy w/Augmentation	59	51.8
Reduction mammoplasty w/Augmentation	25	21.9
Revision (secondary)	89	43.2
Revision (>2)	3	1.5
Revision augmentation	29	31.5
Revision Mastopexy w/Augmentation	55	59.8
Revision reduction mammoplasty w/augmentation	8	8.7
Implant Use		
Silicone gel (smooth)	191	93
No implant	15	7
Implant volume cc (SD)	380.6 (144.6)	
Implant/mesh placement		
Subfascial	97	47.1
Submuscular	109	52.9
TIGR matrix use		
Bilateral	201	97.6
Unilateral	5	2.4
TIGR matrix size (cm)		
10 × 15	1	0.5
15 × 20	103	50.0
20 × 30	102	49.5

**Table 3:** Follow-up Distribution (206 Patients)

Follow-up schedule	Days postsurgery	n	%
1 week	1-10	1	0.5
2 weeks	11-28	2	1.0
6 weeks	29-66	5	2.4
3 months	67-135	30	14.6
6 months	136-272	71	34.5
1 year	273-547	87	42.2
2 years	548-912	10	4.9

**Table 4:** Outcomes

	By patient (*n* = 206)		By breast (*n* = 407)	
	*n*	%	*n*	%
Complications requiring surgical intervention	10	4.9	18	4.4
Seroma	4	1.9	7	1.7
Animation deformity	5	2.4	10	2.5
Implant displacement	1	0.5	1	0.2

**Figure 3:**
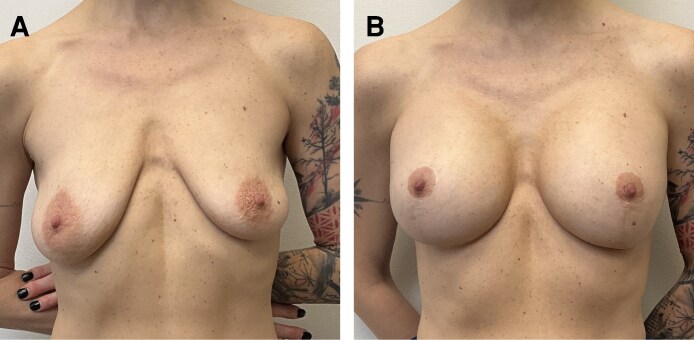
(A) Preoperative images of a 41-year-old female, BMI 20, presenting with micromastia, ptosis, loss of upper pole fullness. Treated with primary submuscular augmentation mastopexy with 280 cc low plus implants and 15 × 20 cm TIGR Matrix bilaterally. (B) Eighteen months postoperative follow-up.

**Figure 4:**
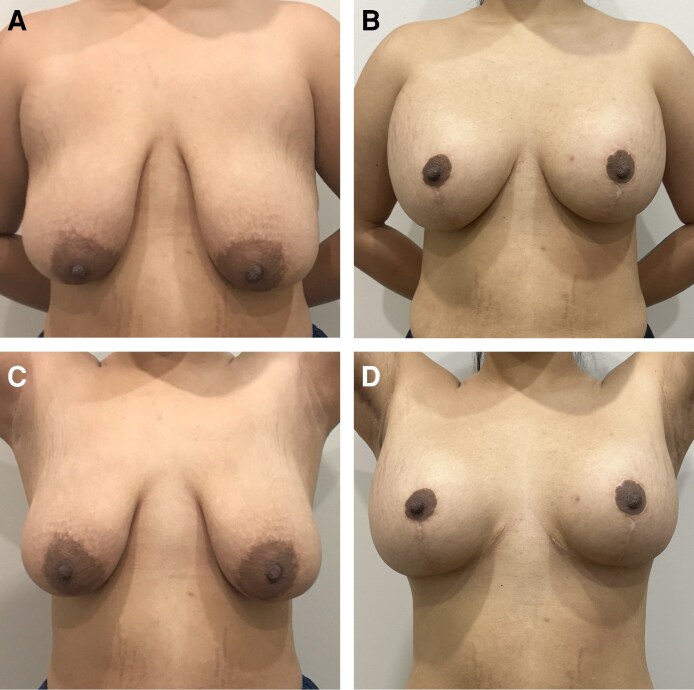
(A, C) Preoperative images of a 28-year-old female, BMI 29.3 following 80-pound massive weight loss, presenting with bilateral macromastia, ptosis, and asymmetry. The patient was treated with reduction (right breast 433 g; left breast 566 g) and primary subfascial augmentation mastopexy with moderate high-profile implants (545 cc right; 500 cc left) and 15 × 20 cm TIGR Matrix bilaterally. (B, D) Two-year postoperative follow-up.

**Figure 5:**
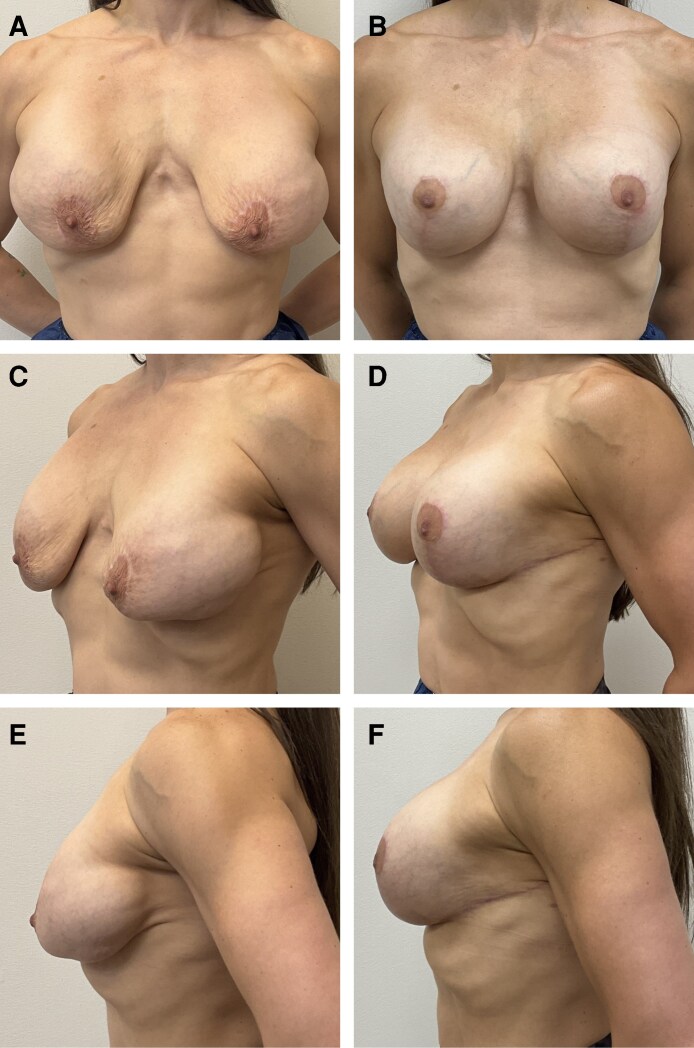
Preoperative (A) frontal, (C) oblique, and (E) lateral views of a 37-year-old female, BMI 23.2, presenting with inferiolateral implant malposition and breast ptosis. Performed revision bilateral mastopexy with bilateral implant exchange with site change from 350 cc saline submuscular implant to 415 cc moderate plus implant placed in the subfascial plane and 15 × 20 cm TIGR Matrix bilaterally. (B, D, F) Postoperative images at 7 months follow-up.

**Figure 6:**
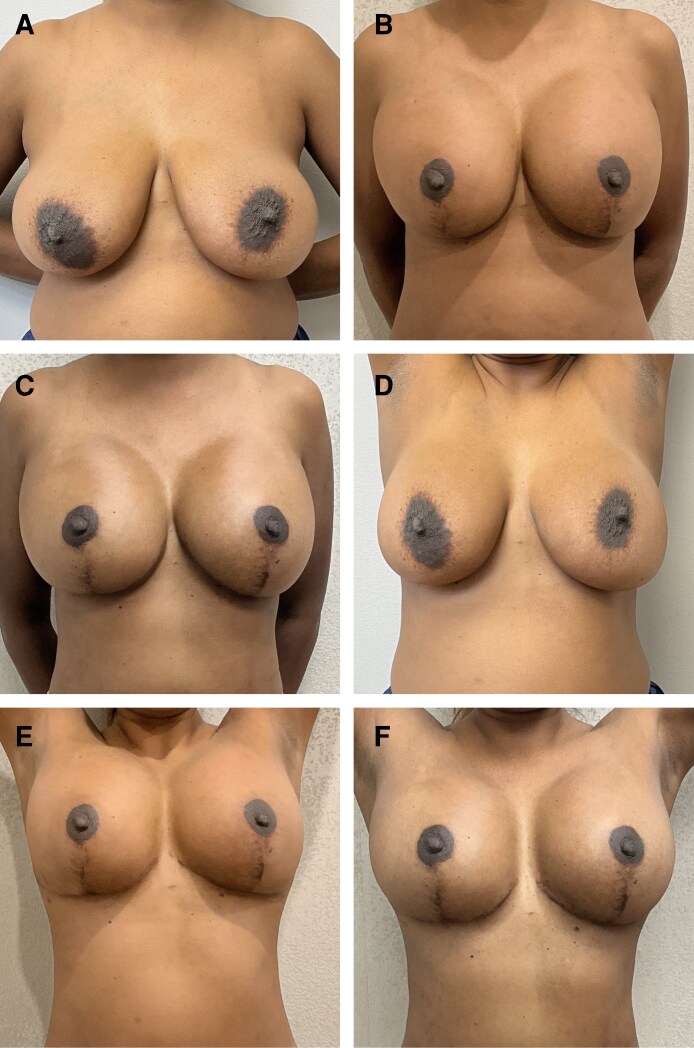
(A, D) Preoperative images of 49-year-old female, BMI 22.6, presenting with macromastia, weighted lower poles and ptosis who desired large implants. A bilateral primary reduction was performed (right—442 g, left—448 g) with bilateral augmentation placing 635 cc moderate high-profile implants submuscular with 15 × 20 cm TIGR matrix bilaterally. (B, E) Postoperative images at 6 months follow-up. (C, F) Postoperative images at 1 year follow-up.

**Figure 7:**
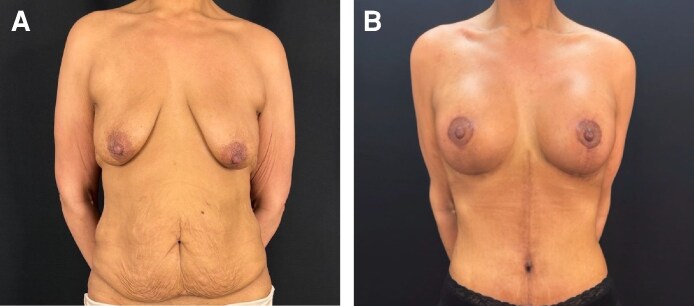
(A) Preoperative images of a 42-year-old female following massive weight loss presenting with macromastia, and ptosis. A bilateral mastopexy with primary augmentation including 15 × 20 cm TIGR matrix bilaterally and Fleur-de-Lis abdominoplasty was performed. (B) Postoperative images at 6-month follow-up.

**Figure 8:**
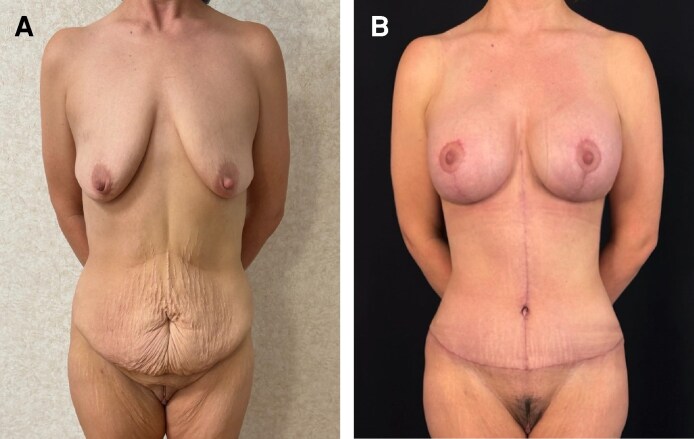
(A) Preoperative images of a 38-year-old female following massive weight loss with extremely fragile tissue. Treatment of macromastia and ptosis with bilateral mastopexy with primary augmentation included 15 × 20 cm TIGR matrix bilaterally. Concurrent Fleur-de-Lis abdominoplasty was performed. (B) Postoperative images at 6-month follow-up.

Figures 3-8 depict representative preoperative and postoperative outcomes among patients returning for follow-up.

## DISCUSSION

The growing role of soft tissue support in aesthetic breast surgery reflects the need for more reliable long-term control of implant position and pocket stability.^13^ Traditional options for soft tissue reinforcement, such as ADMs, offer improved support but are associated with high cost, potential biologic variability, and increased risk of complications, including seroma and infection.^3,5,14^

Synthetic meshes are an appealing alternative to biological matrices offering standardized manufacturing with consistent mechanical properties and predictable resorption profiles. They are designed to provide mechanical support while accommodating concurrent tissue remodeling. In addition, the substantially lower cost of synthetic mesh represents a practical advantage, potentially expanding the applicability of mesh-assisted techniques in aesthetic breast surgery.^15^

TIGR Matrix is a warp-knit, macroporous structure composed of 2 synthetic fibers with distinct resorption profiles. The fast-absorbing fibers compromise 40% of the mesh and resorb within 2 weeks to 4 months, whereas the remaining fibers resorb gradually over a period of 6 to 36 months. Unlike more rapidly absorbable meshes that may lose support prematurely or permanent meshes that carry theoretical risks of chronic foreign-body reaction,^16^ the TIGR Matrix design offers extended structural support of natural tissue regeneration over time, providing strength and stability in the initial wound healing phase, followed by increased elasticity and flexibility of the scaffold as the faster-absorbing copolymer component is resorbed.^12^

Clinically, the material exhibited desirable handling characteristics, including sufficient malleability to permit intraoperative contouring while maintaining stability after fixation. In our series, 4 patients developed 5 postoperative seromas occurring between 10 days and 3 months postsurgery. Three of the 4 presented for breast surgery following substantial weight loss ranging from 62 to 110 pounds. The initial operative approach in 2 patients was primary subfascial augmentation with breast reduction, 1 was primary subfascial augmentation with mastopexy, and 1 was primary submuscular augmentation. In all cases, the seromas were managed with removal of the implant and any nonintegrated mesh and thorough washout of the breast pocket. Two patients returned for subsequent augmentation 4 to 9 months after resolution of the seroma and the other 2 received immediate replacement with a new implant and mesh for soft tissue support. One of these latter patients experienced a recurrent seroma 3 weeks later, which necessitated repeat removal of the mesh and implant. Six months later, she underwent secondary replacement of the implant and mesh. At latest follow-up, all 4 patients were clinically stable with no evidence of further complications.

There were no reported complaints of mesh palpability following placement in this early review timeframe. We believe this may be due to the malleable nature of the mesh and the operative technique of draping the scaffold over the implant anteriorly with use of a urethral dilator to smooth the mesh over the implant. We do not suture the mesh superiorly. If any mesh is folded on itself at the inferior edges, our technique is to trim and close that edge with a 2-0 PDS suture. This obviates any mesh overlap or potentially palpable corners in the inferior pole of the breast.

While our series included 92 revision cases, there were no clinically identifiable capsular contractures seen during the postoperative follow-up period. We believe meticulous surgical techniques including generous irrigation of the breast pocket with hypochlorous acid intraoperatively, minimal handling of the prosthetics, utilization of a funnel for implant insertion and glove changes for both implant and mesh insertion along with a course of routine postoperative antibiotics may play a role in these early positive outcomes. We recognize, however, that these short-term results do not preclude the future formation of capsular contracture.

A slight majority of the patients (55%) in this series were primary surgeries, and the plane of implant or mesh placement was nearly evenly distributed between submuscular (52%) and subfascial (48%). All 5 instances of animation deformity observed occurred in cases with submuscular placement, with 4 of 5 being primary procedures. Each of these cases was subsequently managed with surgical conversion to a subfascial plane, performed between 8 months and 1 year following the primary procedure.

Wong and Hamilton recently published a systematic review summarizing 31 studies and 2425 patients in aesthetic breast surgery using mesh support. Within these studies, 16 specifically investigated use of mesh in breast augmentation or implant-related revisional surgeries. Reported complication rates ranged from 1.6% to 4.8% and included seroma, hematoma, asymmetry, implant malposition, and capsular contracture. The authors reported overall low complication rates, improved lower-pole support, and high patient satisfaction across mesh types but emphasized the need for standardized reporting and prospective comparative data.^4^ Our findings are consistent with their conclusions and provide further support for the safety and practicality of long-term resorbable synthetic matrices in aesthetic settings.

To our knowledge, this 2-surgeon series of 206 women (407 breasts) represents the first dedicated evaluation of TIGR Matrix in purely aesthetic breast surgery and the largest cohort of aesthetic breast surgeries using the long-term resorbable multifilament synthetic mesh. The results of this study suggest that TIGR Matrix may be a useful adjunct in appropriately indicated aesthetic breast procedures. The material exhibited desirable handling characteristics, including sufficient malleability to permit intraoperative contouring while maintaining stability after fixation. Overall complication and reoperation rates were low and consistent with previously reported outcomes for other synthetic, resorbable scaffolds. Importantly, the use of TIGR Matrix supported the achievement of intended aesthetic outcomes in this patient population through early term follow-up.

When selecting from the array of options of mesh materials, surgeons and patients must consider material handling, biocompatibility, tissue integration characteristics, and safety profile. Over the past decade, both authors have gained extensive experience using biologic and other permanent and resorbable synthetic meshes before adopting TIGR Matrix as our preferred choice when a supportive mesh is indicated in aesthetic breast surgery. We routinely use the matrix prophylactically in primary augmentation and mastopexy-augmentation performed in the subfascial plane, as well as in submuscular cases characterized by compromised soft-tissue quality, limited tissue coverage, or the use of large-volume implants. In addition, we incorporate TIGR Matrix into the surgical plan for almost all revision augmentation and mastopexy-augmentation procedures. Within our practices, the mesh is considered an integral component of these aesthetic cases and included in the overall cost of service. We recognize, however, that ultimately material selection is individualized based on patient and surgeon preference, native tissue quality, and cost considerations, which may vary across practice settings.

Limitations of this review include the lack of a comparator group and the heterogeneous mix of procedures included in our cohort. While follow-up was sufficient to evaluate safety and early outcomes, longer-term follow-up beyond 3 years is necessary to assess outcomes after TIGR Matrix's slow degrading fibers have fully resorbed. We plan to continue to follow these patients through the 5-year postoperative period and expect to publish longer-term results when available. Additionally, our routine practice does not include administration of standardized patient questionnaires or blind assessors at patients’ follow-up visits. Thus, we recognize there may have been detection bias within this retrospective review. Future prospective randomized studies evaluating TIGR Matrix compared to other meshes which include validated patient reported outcomes tools and observational evaluations performed by independent clinicians at follow-up visits would be helpful to further characterize the product's safety, efficacy and utility.

## CONCLUSIONS

TIGR Matrix demonstrated a safety profile comparable to that reported for biologic and other resorbable synthetic meshes in aesthetic breast surgery. Its ease of handling and consistent early performance across primary and revision cases, in both the subfascial and submuscular planes, supports its role as a viable alternative for soft-tissue support in aesthetic breast surgery.
